# The mosquitoes (Diptera: Culidae) of Seychelles: taxonomy, ecology, vectorial importance, and identification keys

**DOI:** 10.1186/1756-3305-5-207

**Published:** 2012-09-21

**Authors:** Gilbert Le Goff, Philippe Boussès, Simon Julienne, Cécile Brengues, Nil Rahola, Gérard Rocamora, Vincent Robert

**Affiliations:** 1 MIVEGEC Maladies infectieuses et vecteurs : écologie, génétique, évolution et contrôle (UMR IRD 224, CNRS 5290, UM1, UM2), Centre IRD France-Sud, BP 64501, Montpellier cedex 5, 34394, France; 2 Ministry of Health, Victoria, Mahe, Seychelles; 3 Island Conservation Society, PO Box 775, Pointe Larue, Mahé, Seychelles

**Keywords:** The Seychelles, Mosquito, Culicinae, Alphataxonomy, Identification key, Biodiversity, Biogeography, Island

## Abstract

**Background:**

During recent periods, the islands of the Republic of Seychelles experienced many diseases such as dengue, chikungunya, Bancroft’s filaria and malaria. Mosquitoes transmit the agents that cause these diseases. Published information on mosquitoes in the Seychelles is notably dispersed in the literature. The maximum number of species obtained on a single field survey does not exceed 14 species.

**Methods:**

We performed a comprehensive bibliographic review using mosquito and Seychelles as the key words, as well as conducted a mosquito field survey for larval and adult stages during the rainy season in December 2008. Sixteen sites were sampled on four granitic islands (Mahé, Praslin, La Digue and Aride) and six sites on coralline atolls in the extreme southwest of the country (Aldabra group).

**Results:**

We found published references to 21 mosquito species identified at least on one occasion in the Seychelles. Our collections comprised 18 species of mosquitoes, all of them from the subfamily Culicinae; no Anophelinae was found. We also confirm that *Aedes seychellensis* is a junior synonym of *Ae.* (*Aedimorphus*) *albocephalus*. The first records for *Culex antennatus* and *Cx. sunyaniensis* are presented from the country, specifically from Aldabra and Praslin, respectively. Based on a comparison of the taxa occurring on the granitic *versus* coralline islands, only three species, *Ae. albocephalus*, *Cx. scottii* and *Cx. simpsoni* are shared. *Aedes albopictus* appeared to exclude largely *Ae. aegypti* on the granitic islands; however, *Ae. aegypti* was common on Aldabra, where *Ae. albopictus* has not been recorded. The notable aggressiveness of mosquitoes towards humans on coralline islands was mainly due to two species, the females of which are difficult to distinguish: *Ae. fryeri* and *Ae.* (*Aedimorphus*) sp. A. The number of mosquito species collected at least once in the Seychelles is now 22, among which five species (*Ae.* (*Adm*) sp. A, *Cx. stellatus*, *Uranotaenia browni*. *Ur. nepenthes* and *Ur. pandani*) and one subspecies (*Ae. vigilax vansomerenae*) are considered as endemic. Two illustrated identification keys, one for adult females and the other for larval stages, are presented.

**Conclusions:**

The knowledge of the culicidian fauna in the Seychelles has been notably updated. The number of mosquito species is relatively large with regards to land surface and distances to continental Africa, although the anophelines are totally lacking. The complex natural history of mosquitoes in the Seychelles provides examples of both vicariance- and dispersal-mediated divergences. They present superb examples for theoretical and applied island biology.

## Background

Mosquitoes of the family Culicidae are distributed worldwide and comprise more than 3500 species. The present paper focuses on the mosquitoes present in a part of the Indian Ocean, namely the Republic of Seychelles. We summarise what is already known about Seychellois mosquitoes based on a literature survey, as well as the original results obtained during a field entomological survey in December 2008, during the north-west monsoon season. This field survey did not find any mosquito from the Anophelinae subfamily and this absence motivated the first scientific paper associated with these results [1]. The absence of anophelines in this large tropical area, certainly with a suitable climate, is striking but has important implications in terms of public health and for local economy sectors depending on tourism. Specially, the Seychelles are malaria free, which constitutes a unique situation in the entire tropical Indian Ocean. Published information on mosquitoes in the Seychelles is notably dispersed and the maximum number of taxa obtained on a single field survey does not exceed 14 species. Syntheses on Seychellois mosquitoes are available, with the maximum number of taxa not exceeding 19 species [2].

### Study area

#### Geography and climate

**Figure 1:**
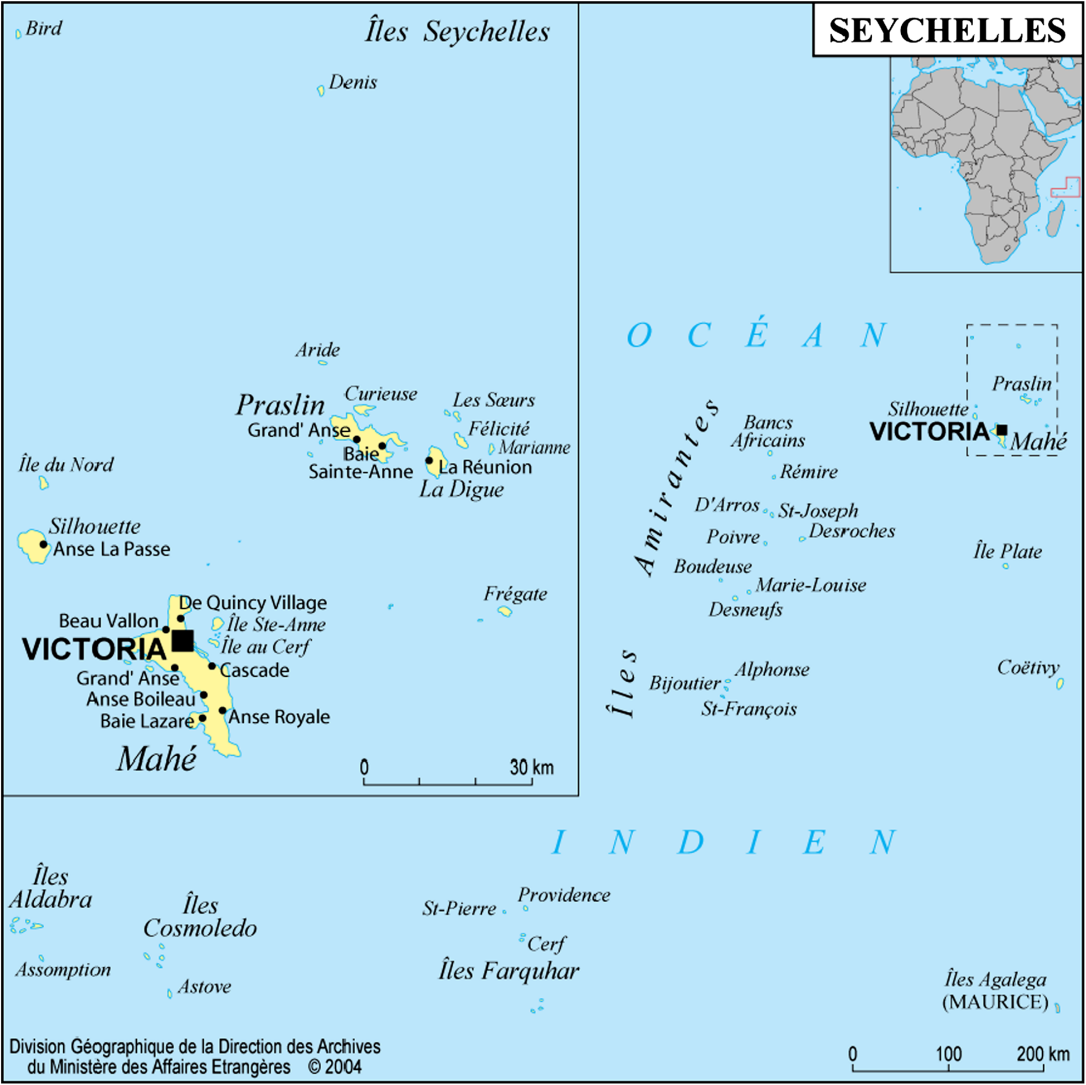
** Map of the Republic of Seychelles in the south-west Indian Ocean.** Seychelles comprise several distinct archipelagos and islands.

A detailed overview of the Seychelles has been presented elsewhere [1]. Briefly, this country is an island state of the Indian Ocean composed of about 115 islands or islets grouped in several distant archipelagos (Figure 1). The largest island, Mahé, hosts the capital city, Victoria, and the highest peak in the country, Morne Seychellois, at 930 m. The Seychellois islands can be divided in two distinct types concerning their geological substrate, granitic and coralline, which also differ with regards to their human colonization history, elevation and climatic regime. The granitic islands are located in the north-eastern part of the Seychelles, about 1,000 km northeast of Madagascar and 1,500 km east of Kenya. These islands have been separated from other emerged land for c. 75 million years, and subsequently have never been totally submerged under the sea. 

A peculiar and very interesting mosquito habitat is the Vallée de Mai on Praslin Island; this high valley is a preserved primary palm forest dominated by the famous Coco-de-mer (*Lodoicea maldivica*), other endemic palms and screwpalms (*Pandanus* spp). Coralline islands have a maximum elevation above sea-level of a few meters and, consequently, they were totally submerged during periods of higher sea-level in recent geological history. For instance, the Aldabra Atoll was completely underwater on at least two occasions, with the most recent complete submersion occurring c. 125,000 years ago [3-5]. The climate on the granitic islands is sub-equatorial (for instance in Victoria: year round humidity over 80%, mean annual rainfall of 2.7 m, temperature from 24 to 30°C), while the conditions are more tropical with a marked dry season in coralline islands (for instance in Aldabra: dry season from April to October, mean annual rainfall of 1.1 m, temperature from 22 to 32°C).

#### Mosquito borne diseases in the Seychelles

Many mosquito-transmitted diseases, including arboviroses, Bancroft’s filaria (*Wuchereria bancrofti*) and malaria have been reported in the Seychelles. The first dengue-like epidemic was observed in the country in 1926–1927. A second event occurred in 1976–1977 when the Seychelles was struck by an extensive epidemic of dengue type 2, with *Aedes albopictus* being the vector. No cases of hemorrhagic fever and shock syndrome associated with this disease have been reported from the country. Prevalence of antibodies collected after the epidemic indicated that approximately 60% of the population had been infected in Mahé [6,7]. Rare sporadic cases of dengue have been reported during the following decades, all without hemorrhagic fever or shock syndrome [8].

No clinically diagnosed cases for the viruses Chikungunya, Sindbis, West Nile and Wesselsbron have been reported in the granitic islands during the 20^th^ century, despite the fact that positive serology was found for Chikungunya and Sindbis [9] and West Nile [7]. From July 2005 to late 2007, the Seychelles experienced epidemics of Chikungunya with *Ae. albopictus* as the presumed main vector. After the epidemic, 60% of the human population had been in contact with the virus [10].

The earliest written record of filariasis in the Seychelles seems done in 1835 by James Holman in *A voyage round the world*: "It is a very healthy climate, and of diseases, hydrocele, erysipelas, and in a few instances hydrothorax, are the most prevalent" [11]. Some decades later, the 1866 Civil Commissioner Report mentions: "The majority of the inmates at Ile Curieuse are not lepers, but are suffering from elephantiasis, hydrocele […], unfortunately common among these people" [12]. The 1926 Annual Report of the Medical Department, which lists four cases [13]. Elephantiasis is listed in the Annual Report of the Medical Department for 1931 as occurring in "the outlying districts" that correspond nowadays to the far south and south-west Seychelles islands (Providence-Farquhar and Aldabra groups, respectively). The 1934 report mentions, "Cases of elephantiasis of the legs and the scrotum are fairly common and many cases of lymphadenitis, lymphangitis and hydrocele seen here are probably of filarial origin". In 1967, a night-blood survey of microfilaraemia by Frölich [14] revealed an overall level of infection at 4%, reaching 20% in Port Glaud, on Mahé, and 17% in Praslin. In the vector, nine natural infections were found in 429 *Culex quinquefasciatus* (= 2.1%) collected at Port Glaud, with one mature *Wuchereria bancrofti* larvae [13]. Natural infections and experimental transmission showed that *Cx. quinquefasciatus* is the main and probably only vector in the Seychelles.

A serologic survey focused on Brancroft’s filaria with sera from native people randomly selected among the outpatients of the Victoria General Hospital. A 17% infection rate was reported in the Seychelles population in 1972 [15]. A 3.6% microfilaria positive rate was detected in 1979 [16]. Epidemiological records of the recent years indicate no clinical incidence of filarial and its transmission appears to have completely ceased [8]. Dog filariosis due to *Dirofilaria immitis* is common in the Seychelles, with *Aedes* mosquitoes among which *Ae. albopictus* as vectors; this nematode does not develop in humans.

Annually, approximately 10 cases of malaria are reported from the country — all imported and mostly in expatriate Indian workers — as the Seychelles are free of anopheline mosquitoes [1,15]. The only cases of native malaria in the Seychelles were reported during two epidemics – one in 1908 from Aldabra for the benign tertian type, and the other in 1930–31 from Aldabra and Assomption islands for *Plasmodium falciparum*[17]. The presence of *Anopheles gambiae s.l.* was documented [18] and constitutes the unique records for anophelines in the Seychelles. This introduction of anophelines to these islands was apparently not followed by their successful colonization over the following dry season [1]. The observation of anophelines on Assomption Island in 1975 [19] at larval stage is probably an accidental pipette contamination [1], as confirmed by the absence of symptoms suggesting malaria among the hundred or so inhabitants and the absence of anopheline in 1977 [20].

In 1968–69, entomological observations were carried out over a period of 11 months on 14 islands in the country [15] (Table 1). Metselaar *et al.*[6] indicated 14 mosquito species in Mahé and nearby islands (*i.e.* granitic) but without giving the precise taxonomic list. Bin *et al.*[21] identified 16 mosquito species on the granitic islands and this list is summarised in Yersin *et al.*[8]. More recently, Gerlach [2] concluded that 19 mosquito species occur in the Seychelles and provided information on their distribution, ecology and identification. 

**Table 1:** List of 21 mosquito species reported from different studies on mosquitoes in the Seychelles, before 2008

**Genus**	**(Subgenus)**	**Species and subspecies (if any)**	**Theobald 1912 **[22]	**Hermitte 1931 **[17]	**Edwards 1941 **[23]	**Harper 1947 **[24]	**Mattingly & Brown 1955 **[25]	**Lambrecht 1971 **[15]	**Van Someren 1972 **[26]	**Gerbert & Anett 1976 **[27]	**Bin*et al.*1996 **[21]
*Aedes*	*Aedimorphus*	*albocephalus*					G	G		G	
	*Aedimorphus*	seychellensis*	G Al		G Al		G				
	*Aedimorphus*	*species A*							Al		
	*Coetzeemyia*	*fryeri*	Al		Al Co				Al		
	*Ochlerotatus*	*vigilax vansomerenae*					G	G		G	
	*Stegomyia*	*albopictus*	G Am		G	G	G	G		G	G
	*Stegomyia*	*aegypti*	G Am		G	G		G			G
	*Skusea*	*lambrechti*				G	G	G		G	
*Anopheles*	*Cellia*	*gambiae s.l.*		Al As							
*Culex*	*Culex*	*fuscocephala*									G
	*Culex*	*quinquefasciatus*	G		G	G	G	G		G	G
	*Culex*	*scottii*	G		G		G	G			
	*Culex*	*simpsoni*				G	G	G		G	
	*Culex*	*sitiens*					Al				
	*Culex*	*tritaeniorhynchus*									G
	*Eumelanomyia*	*stellatus*				G	G	G		G	G
	*Eumelanomyia*	*wigglesworthi*						Vm			
*Mansonia*	*Mansonioides*	*uniformis*	G				G				
*Uranotaenia*	*Pseudoficalbia*	*browni*					Vm	G		G	
	*Pseudoficalbia*	*nepenthes*	G		G	G	G	G		G	
	*Pseudoficalbia*	*pandani*	G		G	G	G	G		G	G
Total			9	1	8	8	14	13	2	10	7

* The synonymy, previously suspected [23], between *Ae. albocephalus* and *Ae. seychellensis*, is demonstrated in the present article.

G = granitic islands; Vm = Vallée de Mai on Praslin; Am = Amirantes Islands; Al = Aldabra Atoll; As = Assomption Island; Co = Cosmoledo Atoll.

In order to provide an updated synthesis on mosquito populations in the Seychelles, including insight into patterns of mosquito species richness, biology and colonization/extinction patterns, we conducted a field survey of several different granitic and coralline islands, the results of which are presented here. These findings are then placed in the framework of a comprehensive survey of published and unpublished information on Seychellois mosquitoes, which is not extensive, but notably dispersed in the literature.

## Methods

Information on the mosquitoes of the Seychelles was collected from the scientific literature. Further, we had access to a number of unpublished reports available within the country.

A field survey was conducted from 29 November to 18 December 2008, during the rainy season, when the mosquito densities are assumed to be high. The islands chosen for the survey were based on the following rationale. Four granitic islands were selected, including the three most populated (Mahé, Praslin, La Digue) and a small granitic island (Aride). Four coralline islands belonging to the Aldabra group were selected (Picard, Malabar, and Grande Terre within the Aldabra Atoll, plus neighbouring Assomption). The list of visited islands and precise details of sampled sites are presented in Table 2.

**Table 2:** Species identification for adult (A) and larval (L) Culicinae mosquitoes collected during the 2008 field survey in the Seychelles

**Island**	**Localities of collections (and Nb of CDC light-trap x night)**	***Ae. albocephalus***	***Ae. (Adm.)*sp A**	***Ae. fryeri***	***Ae. vigilax vansomerenae***	***Ae. albopictus***	***Ae. aegypti***	***Ae. lambrechti***	***Cx. antennatus***	***Cx. quinquefasciatus***	***Cx. scottii***	***Cx. simpsoni***	***Cx. sitiens***	***Cx. tritaeniorhynchus***	***Cx. stellatus***	***Cx. sunyaniensis***	***Ur. browni***	***Ur. nepenthes***	***Ur. pandani***	**Species richness**
Mahé	Victoria (2)									A										1
	Morne Blanc (0)					A^a^														1
	Tea plantation (0)																	L		1
	Copolia (6)									A									A	2
	Petit Paris (2)									A										1
	Anse Intendance (2)									A		A		A						3
	Baie Police (2)											A		A						2
	Baie Lazare (2)					A						A			A				A	4
	Port Launay (2)	A						A												2
Aride	(6)											AL								1
Praslin	Anse Kerlan (6)					A		A		A										3
	Vallée de Mai (3)	A						A		A	A	A				A	AL		AL	8
	Fond B’Offay (1)	A								A										2
	Côte d’Or (0)					L		A^a^		L		L		A^b^L						5
	Marie Jeanne (1)					A		A			A			A						4
	Grande Anse (1)	A				L				AL				A						4
	Airport (0)					A^a^L				L		L								3
La Digue	La Passe (4)	A				A				A		A							A	5
	La Veuve Réserve (2)	A				L				A	A	A		A				A	L	8
	L’Union (6)	A			A			A		A		AL								5
Aldabra-Picard	Research station area (9)	A^a,b^	AL	A^b^			A^b^L					A	A							6
Aldabra-Malabar	Middle camp (4)	A^a^	A^a^L	A^a^L			A^a,b^L						AL							5
Aldabra-Grande Terre	Takamaka Grove (4)	A^a^	A^a^L				A^a,b^L		A											4
	Cinq Cases (4)		AL	A^a^			A					L								4
	Anse Maïs (3)		A	A			A		A		A		A							6
Assomption	Houses and airport (4)		A	A								AL								3
Total of positive sites		10	6	5	1	9	5	6	2	13	4	13	3	6	1	1	1	2	5	93

^a^ Some adult mosquito recorded alighting on and biting humans.

^b^ Some adult mosquito recorded alighting on and biting Aldabra tortoises.

Most adult mosquitoes were collected using CDC light-traps. *Culex antennatus* and *Culex sunyaniensis* were previously unrecorded in the Seychelles.

Entomological surveys included three main methods. (1) Detailed examinations for immature stages (larvae and pupae) were performed in stagnant or slowly running water, at natural and human-made water collection sites (marshes, tree holes, crab holes in mangroves, containers and reservoirs, etc.). Larval sampling was performed in a given area at all sites with collected water, but the less accessible house gutters, tree-holes, rockpools or underground pools being undersampled. (2) Adult mosquitoes were collected using CDC Miniature Light Trap 6 volts (BioQuip™) from dusk to dawn, with a 4-watt incandescent light and a 4-watt black light tube (UV light ca. 320–420 nm). (3) On occasions, alighting mosquitoes on members of the field team were collected. Also, on Aldabra, mosquitoes were collected biting Aldabra tortoises (mainly on their posterior legs).

In the laboratory, all male genitalia and larvae were mounted on slides in Euparal before microscopic examination.

DNA extraction for molecular work aimed to study *Aedes* of the coralline islands. Individual mosquitoes (adult or larvae) were ground in 200 μl of 2% CTAB, which was left 5 min at 65°C, after which 200 μl of chloroform was added and mixed gently. After centrifugation (12,000 rpm, 5 min), the upper phase was collected and 200 μl of isopropanol was added. The mix was centrifuged for 15 min (12,000 rpm) and the isopropanol was drained off. An extra step of ethanol 70% was carried out to purify the DNA. After the removal of the ethanol, the DNA was dried using a speed-vac and finally eluted with 20 μl of water. The extracted DNA was used as the template for the amplification of the mitochondrial NADH deshydrogenase subunit 4 (ND4) gene (ND4F: 5’-ATTGCCTAAGGCTCATGTAG-3’ and ND4R: 5’-TCGGCTTCCTAGTCGTTCAT-3’) [28] and the ribosomal gene ITS2 (5.8 s: 5’-TGTGAACTGCAGGACACATG-3’ and 28 s: 5’-ATGCTTAAATTTAGGGGGTA-3’) [29]. Each PCR was performed in a 25 μl final volume. The PCR mix was the same for all the primers used, with 4 μl of DNA extraction (diluted at 1/50), 1X Buffer (QIAGEN), 1.5 mM of MgCl_2_, 0.2 mM of each dNTP mix (5 mM), 10 pMole of each primer and 1 unit of Qiagen Taq Polymerase (5 u/μl). The PCR amplification program was the same for the primers except the annealing temperature differed: after 2 min of denaturation at 94°C, 35 cycles of 30 s denaturation at 94°C, 30 s annealing (54°C for ND4; 52°C for ITS2) and 1 min extension at 72°C, followed by 10 min final elongation at 72°C. PCR products were purified using AMPure PCR kit (Agencourt, Beverly, MA).

Sequencing of amplified fragments was carried out on a single strand by using the ABI Prism BigDye terminator version 3.1 (Applied Biosystems). Each 10 μl reaction contained 1 μl of Ready reaction mix (Applied Biosystems), 1 μl of 5X sequencing buffer, 5 pMole of primer (ND4F, ND4R, 5.8 s or 28 s) and 1 μl of purified PCR product. After an initial denaturation step at 96°C for 1 min, 25 cycles of 10 s at 96°C, 10 s at 50°C and 3 min at 60°C were performed. Sequence reactions were purified using SeqClean kit (Agencourt) and analysed on the ABI 3130XL automatic sequencer (Applied Biosystems). Electrophoregrammes were obtained, checked and sequences aligned with Bioedit 7.0.9.0. [30] using ClustalW algorithm.

The statistical analysis of mosquito number collected in traps using UV light as compared to those with incandescent light was performed using a one-level mixed model with a random intercept to take into account dependency between the counts of mosquitoes in traps at the same night and collection site, and using a negative binomial mixed effect regression to take into account the over dispersion of the data with the count of mosquitoes in each trap as the dependant variable and the type of trap as the independent variable.

Two illustrated identification keys, one for adult females and the other for larval stages, are presented ( Additional file 1). We used biological materials collected during our field survey and specimens in the IRD collection “Arthropods of medical interest” Montpellier, France.

Throughout this paper, we maintain usage of the traditional taxonomy of mosquitoes ( http://www.mosquitocatalog.org/), and do not follow the newly proposed classification of Aedini (see Clements [31]).

## Results

### Twenty two mosquito species collected at least once in the Seychelles

**Figure 2:**
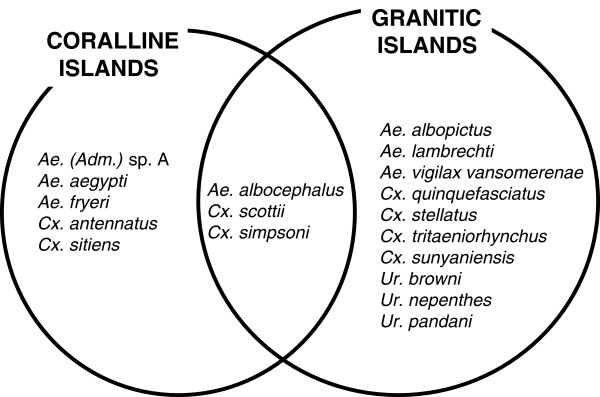
List of the 18 mosquito species observed on the granitic and/or coralline Seychelles during our field survey in December 2008.

Published literature before 2008 contains the mention of 21 species recorded at least once in the Republic of Seychelles (Table 1). *Anopheles gambiae s.l.* was observed in the Aldabra Atoll and on Assomption Island as a non-resident species in 1930 [1,17], and the presence of an unnamed *Aedes* belonging to the subgenus *Aedimorphus*, hereafter referred to as *Ae.* (*Adm.*) sp. A has been mentioned from Aldabra [26] (see text below). Our field study in late 2008 resulted in the collection of 18 species (Figure 2), all of which has been previously reported from the Seychelles, with two notable exceptions – *Cx. antennatus* in Takamaka and Anse Maïs (two sites on Grande Terre, Aldabra) and *Cx. sunyaniensis* in Vallée de Mai (Praslin). In addition, the suspected synonymy between *Ae. albocephalus* and *Ae. seychellensis* is herein confirmed and we provide the first gene sequencing for Seychellois specimens of this species. With these new results, the number of mosquito species collected at least once in the Seychelles is 22 (see Table 3, with details on taxonomy and distribution). The following list of these species is ranked by alphabetic order, with 7, 1, 10, 1 and 3 species belonging to the genera *Aedes*, *Anopheles*, *Culex*, *Mansonia* and *Uranotaenia*, respectively. 

**Table 3:** Comprehensive list of the 22 mosquito species observed at least once in the Seychelles between 1903 and 2008

**Sub-family**	**Genus**	**Subgenus**	**Species and subspecies (if any)**	**Species descriptor and year of description**	**Area of distribution**	**First mention in the Seychelles**
Anophelinae	*Anopheles*	*Cellia*	*gambiae s.l.*	Gilles 1902	Tropical Africa, Madagascar, Comoros, Mauritius, La Réunion	[17]
Culicinae	*Aedes*	*Aedimorphus*	*albocephalus*	(Theobald 1903)	Tropical Africa, Madagascar, Comoros	[22]
		*Aedimorphus*	species A	to be fully described and named	Endemic to the coralline Seychelles	[26]
		*Coetzeemyia*	*fryeri*	(Theobald 1912)	East coast of Africa, Madagascar	[22]
		*Ochlerotatus*	*vigilax vansomerenae*	Mattingly 1955	Species in south-east Asia, Indonesia and Australia; subspecies endemic to the granitic Seychelles	[25]
		*Stegomyia*	*albopictus*	(Skuse 1895)	From Japan to India, plus Madagascar; now invasive in many areas of the world	[32]
		*Stegomyia*	*aegypti*	(Linnaeus 1762)	Pantropical	[33]
		*Skusea*	*lambrechti*	van Someren 1971	The granitic Seychelles, Madagascar	[24]
	*Culex*	*Culex*	*antennatus*	(Becker 1903)	Africa (Angola, Botswana, Sao Tome and Principe, Algeria, Tunisia, Egypt), Middle East, Madagascar, Mayotte	The present article
		*Culex*	*fuscocephala*	Theobald 1907	Asia	[34]
		*Culex*	*quinquefasciatus*	Say 1823	Pantropical	[32]
		*Culex*	*scottii*	Theobald 1912	The Seychelles, Madagascar	[22]
		*Culex*	*simpsoni*	Theobald 1905	Africa, Comoros, Madagascar	[24]
		*Culex*	*sitiens*	Wiedemann 1828	Africa, Middle East, Asia, Oceania	[25]
		*Culex*	*tritaeniorhynchus*	Giles 1901	Africa, Middle East, Asia	[34]
		*Eumelanomyia*	*stellatus*	van Someren 1947	Endemic to the granitic Seychelles	[35]
		*Eumelanomyia*	*sunyaniensis*	Edwards 1941	Tropical Africa	The present article
		*Eumelanomyia*	*wigglesworthi*	Edwards 1941	Tropical Africa	[15]
	*Mansonia*	*Mansonioides*	*uniformis*	(Theobald 1910)	Africa, Asia, Oceania	[25]
	*Uranotaenia*	*Pseudoficalbia*	*browni*	Mattingly 1955	Endemic to the granitic Seychelles	[25]
		*Pseudoficalbia*	*nepenthes*	(Theobald 1912)	Endemic to the granitic Seychelles	[22]
		*Pseudoficalbia*	*pandani*	(Theobald 1912)	Endemic to the granitic Seychelles	[22]

*An. gambiae s.l.* is considered as a non-resident mosquito in the Seychelles.

### *Aedes* (*Aedimorphus*) *albocephalus*

This species was described (as *Stegomyia albocephala*) from one male bred from a larva found in the Gambia [33]. Then *Ae. seychellensis* was described (as *Reedomyia seychellensis*) from five females collected on both granitic and coralline islands [22]; the later present small narrow-curved scales at the posterior border of the scutellum. This morphological character was not considered as striking by Edwards who observed “scutellum [of *Ae. albocephalus* with broad flat white scales on lateral lobes and sometimes also on median lobe, but more usually the median lobe has some or even all of its scales narrow” and put the two species in synonymy [23]. However he notes: Seychellois “specimens are all females and confirmation of their identity is needed”. This species was further collected on the granitic islands [15,21,27], in tropical Africa and Madagascar (Table 3). In 2008, we collected adult specimens of this species on both granitic (Mahé, Praslin, La Digue) and coralline (Aldabra Atoll) islands, but we did not found the larval stage. The distribution of this African species is large, encompassing much of the area between Gambia, southern Africa and Seychelles [36]. Females are anthropophilic for their blood meals. Breeding sites where larvae have been found include a variety of pools formed by the collection of rainwater, crab holes, inland rockpools, bamboo stumps, abandoned or uncovered canoes and watertanks [15]. In line with Edwards, several authors have agreed that *Ae. seychellensis* is a synonym of *Ae. albocephalus*[8,15,21,25], while others have listed the two species separately [2,37]. In 2008, we found (1) morphological homogeneity in all specimens examined (2 males and 34 females collected on granitic and coralline islands) but inconstant presence of the small narrow-curved scales at the posterior border of the scutellum (2) the male genitalia typical of *Ae. albocephalus*[23,36] and (3) 100% homogeneity in gene sequencing (4 females for NDH4 and ITS2 with 355 bp and 318 bp, respectively [GenBank: JX282415 to JX282422]) both in granitic and coralline islands. Due to this set of arguments, we confirm the synonymy between the two taxa. We collected *Ae. albocephalus* biting indiscriminately humans and giant tortoises on Picard in the Aldabra Atoll (Table 2).

### *Aedes* (*Adm.*) species A and *Ae.* (*Coetzeemyia*) *fryeri*

Due to historical reasons, these two species are presented here together. Theobald described *Aedes fryeri* (as *Culiselsa fryeri*) from 9 females collected in Takamaka, Aldabra [22]. Hopkins described the larva, although he expressed some doubt whether the available larva was actually of *Ae. fryeri*[38]. Indeed, the larva was not of *Ae. fryeri*, which lead to some confusion until van Someren demonstrated that they belonged to an undescribed *Ae.* (*Adm.*) [39], which remains undescribed. Herein we refer to this species as *Ae.* (*Adm.*) sp. A. The morphology of females of these two species is quite similar, except for a dorso-median white band on abdominal tergums VI and VII present in *Ae. fryeri* and absent in *Ae.* (*Adm.*) sp. A, but male genitalia and larva chetotaxie exhibit a number of morphological differences. Mattingly in 1963 [40] (*i.e.* before the van Someren’s observation) described briefly the larval stage of *Ae. fryeri* (as *Ae. mombasaensis*, a taxon later put in synonymy with *Ae. fryeri*[26]) and the larval stage of *Ae.* (*Adm.*) sp. A (as *Ae. fryeri*). *Aedes fryeri* was found in Aldabra Atoll and Cosmoledo Island [23].

In 2008, we collected specimens of *Ae. fryeri* and those of *Ae.* (*Adm.*) sp. A, and, using molecular techniques based on gene sequences (NDH4 and ITS2) we demonstrated the correspondence of female and larval stages for each species. A description of *Ae.* (*Adm.*) sp. A, endemic to the southern (coralline) islands of Seychelles, is in preparation (Boussès P., pers. com.). In the Seychelles, both species are limited to coralline islands, where they can occur in remarkable densities in the rainy season and are exceedingly aggressive to humans during the day. *Aedes fryeri* was collected biting Aldabra tortoises on Aldabra-Picard (Table 2). Larval breeding sites for both species are rock pools, with varying levels of concentration of sodium chloride. Based on current information, *Ae.* (*Adm.*) sp. A has an area of distribution limited to coralline Seychelles, while *Ae. fryeri* is also found on the east coast of Africa and Madagascar (Table 3). Depending on the taxonomic treatment, *Ae. fryeri* has also been placed in the genera: *Culiselsa, Ochlerotatus* and *Levua.* Recently, Huang removed *Ae. fryeri* from the subgenus *Levua* Stone & Bohart (genus *Levua* of Reinert *et al.*[37]) to the new monotypic subgenus *Coetzeemyia*[41]*.*

### *Aedes* (*Ochlerotatus*) *vigilax vansomerenae*

This species is mainly found in the Oriental and Australasian regions but the subspecies *vansomerenae* is confined to the Seychelles. The larval stages are very similar to those of *Ae. fryeri*. Mattingly and Brown identified *Ae. vigilax* for the first time in the Seychelles based on collections from Silhouette and Denis Islands [25]. After these observations, this taxon has been collected regularly on the granitic islands [15,21,27]. In 2008, the unique collection of this taxon was obtained at L’Union, La Digue (Table 2).

### *Aedes* (*Stegomyia*) *albopictus*

This species (*Stegomyia albopicta, sensu* Reinert *et al.*[37]) was observed (as *Stegomyia scutelaris*) on Desroches in 1905 and then on Mahé, Silhouette, Praslin and Denis islands in 1908–1909 [22]. It was then regularly observed on the granitic islands and on the coralline islands of Bird, Denis and Platte [15,21,24,25,27]. Its presence has not been reported on the most southern coralline islands of the Aldabra group. Strikingly, on Mayotte Island, part of the Comoros archipelago, this species — which is easily detected when present — was not observed before and during the 1970s [42], but it has been identified since 2001, with increasing densities between 2007 and 2010 [43,44]. Our 2008 survey, in line with the previous ones, confirms the considerable abundance of this species on the granitic islands, and its absence on Aldabra and Assomption. Interestingly, these latter two islands do not have a proper harbour and are sparsely inhabited by people. *Aedes albopictus* is listed as one of the 100 worst invasive species in the world [45]. Having been identified as the main vector in the Seychelles for several mosquito-born diseases such as Dengue and Chikungunya, it represents a significant threat for public health and the economy of the country [46]. In addition, possible increases in temperature and rainfall associated with climatic change may facilitate the spread of this mosquito and virus transmission [47].

### *Aedes* (*Stg.*) *aegypti*

This species (*Stegomyia aegypti, sensu* Reinert *et al.*[37]) was the first documented in the Seychelles (as *Stegomyia fasciata*), with specimens collected in the Victoria harbour [33]. It was also found on Darros Island in 1905 and Aldabra-Picard in 1908–1909 [22]. A decreasing trend in the density of this species was observed during the second half of the 20th century (first time in 1955 [25], but at normal levels in 1947 [24]). This is probably associated with inter-specific competition with the invasive *Ae. albopictus*, as observed elsewhere in the world [48-50]. Associated with this aspect, certain field surveys have found *Ae. aegypti* in the granitic islands [15,23,24,34], while for others it was absent [25,27]. Nevertheless, since some decades, *Ae. aegypti* is no more considered as a threat for public health due to its low density and limited distribution [21]. In our 2008 study, *Ae. aegypti* was not observed on any granitic island, but was abundant on Aldabra, where it was observed biting humans and Aldabra tortoises (Table 2).

### *Aedes* (*Skusea*) *lambrechti*

This species (*Skusea lambrechti, sensu* Reinert *et al.*[37]) was first reported in the Seychelles by Harper [24], then by Mattingly and Brown [25] (as *Ae. pembaensis*). This mosquito was subsequently described [39] as a valid species endemic to the granitic Seychelles, morphologically similar to *Ae. pembaensis*, which occurs on the east coast of continental Africa. The breeding sites of *Ae. lambrechti* are crab holes. In our 2008 study, *Ae. lambrechti* was observed on Mahé, Praslin and La Digue. This native species cannot be considered as endemic in the Seychelles because it has also been found in the northern coastal area of Madagascar between Nosy-Be and Antalaha [51].

### *Anopheles* (*Cellia*) *gambiae s.l*

The presence of at least one species of the *An. gambiae* complex was reported by Hermitte [17] (as *An. gambiae* (*costalis*)) during the malaria epidemic that occurred from June 1930 to January 1931 on Assomption and then on Aldabra. Because this introduction was apparently followed by the extinction of local populations of this *Anopheles* during the subsequent dry season, it would appear that this species is unable to colonize and establish permanent breeding populations. Therefore, although potentially able to reproduce and spread, anophelines can only be considered as sporadically introduced and certainly not resident in the Seychelles. *Anopheles gambiae s.l.* is the only anopheline recorded in the Seychelles [1].

### *Culex* (*Culex*) *antennatus*

This species has not been previously reported from the Seychelles. Adult females have been collected in a CDC light traps on Aldabra-Grande Terre, in the Takamaka grove (15 Dec 2008) and Anse Maïs (17 Dec 2008) (Table 2). This species is largely distributed in continental Africa and the Middle East; in the Indian Ocean it is known from Mayotte and Madagascar [42] where it is a potential vector of Bancroft’s filaria because it allows complete experimental development of ingested microfilaria [52]. It could be a recent colonizer or a native species that remained undetected by previous surveys.

### *Culex* (*Cux.*) *fuscocephala*

This Asian species was observed only on Mahé and Praslin in 1995 (as *Cx. fuscocephalus*) [21,34]. This species is thought to have been recently introduced to the Seychelles, as an undesirable consequence of human transport activities around the world. It was not recorded during our 2008 survey.

### *Culex* (*Cux.*) *quinquefasciatus*

This species, a typical pantropical urban mosquito, was recorded (as *Cx. fatigans*) from the granitic Seychelles starting in the 20^th^ century [22]. Its apparent rarity, except in the immediate vicinity of the Victoria port, suggests that it is a human introduction [25]. In 1952, this species was also present on Ile Platte [25]. In 1969, it (as *Culex pipiens fatigans*) was one of the most common mosquitoes in the granitic islands [15]. It was regularly found during subsequent entomological surveys conducted on the granitic islands. The only records in the remote coralline islands date from 1973 on Providence and neighbouring Farquhar. These two island groups support very low human populations and are far from any urbanization [53]. During our 2008 survey, this species was found on the granitic islands but not on the coralline ones. We did not visit the Providence-Farquhar group during our 2008 survey.

### *Culex* (*Cux.*) *scottii* and *Cx.* (*Cux.*) *simpsoni*

These species have been regularly found in the past on the granitic Islands. Our 2008 surveys confirm these observations and, in addition, the presence of the two species is documented for the first time in the Aldabra group (Table 2). *Culex scottii* is not endemic to the Seychelles as it is known along the eastern coast of Madagascar near Fenerive-Est [54].

### *Culex (Cux.) sitiens*

This widely distributed species had already been collected on Aldabra in 1907 [25]. We found it on this atoll during the 2008 surveys (Table 2).

### *Culex* (*Cux.*) *tritaeniorhynchus*

This species, an important vector of Japanese B encephalitis in south-east Asia, was collected on Mahé and Praslin in 1995 [21,34]. It is thought to be newly introduced in the Seychelles as an undesirable consequence of transport and trade activities around the world. The presence of this species on Mahé was confirmed in 2008.

### *Culex* (*Eumelanomyia*) *stellatus*

Since 1947, this species has been regularly found in the granitic islands (Table 1). It is considered native and endemic to the granitic Seychelles.

### *Culex* (*Eum.*) *sunyaniensis*

This species was previously unrecorded from the Seychelles. One adult male was collected in a CDC light trap placed in Vallée de Mai, Praslin (5 Dec 2008) (Table 2). The identification was confirmed by dissection of the terminalia. This Afrotropical species is broadly distributed from Senegal to Sudan and Mozambique [55]. At the larval stage this species closely resembles *Cx. wigglesworthi*. However, van Someren was well aware of this similarity, when she described the larval stage of *Cx. wigglesworthi*[56].

### *Culex* (*Eum.*) *wigglesworthi*

The presence of this species is based on a single larva found in a ‘peg hole’ in Vallée de Mai, Praslin, on 3 Dec 1968 and identified by van Someren [15]. Gerlach [2], misinterpreting Gerberg & Arnett [27], wrongly suggests that this species at the larval stage can be confused with *Cx. simpsoni*. Exclusively Afrotropical, *Cx. wigglesworthi* has also been observed on Madagascar and Mayotte [42].

### *Mansonia* (*Mansonioides*) *uniformis*

This species was probably first observed on Mahé [22]. Its presence was later documented on Praslin [25] and is now known from Anse Nord-est (Mahé), a locality with a swamp, where it is aggressive to humans (Pat Matyot, pers. com.). The larval and pupal stages develop attached to the subaqueous parts of water plants. Its distribution encompasses Africa south of the Sahara, Asia and Oceania (Table 3).

### *Uranotaenia* (*Pseudoficalbia*) *browni*

This species is unknown outside of the palm forest habitat of Vallée de Mai, Praslin, with the enigmatic exception of a single female on Mahé [16]. Immature stages develop in leaf axis, fallen leaves and palm rachides. This species is endemic to the Seychelles.

### *Uranotaenia* (*Psc.*) *nepenthes*

This species has been regularly found on the granitic islands. Immature stages appear restricted to the pitchers of *Nepenthes pervillei*. This species is endemic in the Seychelles (see [57] paragraph 2, p. 205) and has curiously adapted to breed in the digestive liquid of carnivorous pitcher plants.

### *Uranotaenia* (*Psc.*) *pandani*

This species has been regularly found on the granitic islands. Immature stages develop in a variety of breeding places such as pools, tanks, palm spadix-sheaths and fallen leaves. This species is endemic to the Seychelles (see [57] paragraph 2, p. 205).

### Quantitative aspects on collecting mosquitoes

Our 2008 study was oriented towards qualitative collections rather than a fine-scale analysis of mosquito catches in quantitative terms. Only notable quantitative aspects are discussed in this section.

The sampling effort with CDC light traps was higher on granitic as compared to coralline islands (48 *vs.* 26 trap-nights, respectively). However, the total number of mosquitoes was much lower on the granitic than coralline islands (1,341 *vs.* 15,066, respectively). Nevertheless, at least one specimen of all the 18 observed species was collected in CDC light-traps (Table 3).

The mean mosquito densities per trap were much higher on coralline than granitic islands (579.5 *vs*. 27.9 mosquitoes per trap-night, respectively, *i.e.* 20.8 times higher; P = 0.0008 by Mann Whitney *U* test).

Although traps using UV light consumed notably more battery power and were more fragile (specifically the light tube) as compared to those with incandescent light, it attracted on average 2.6 times more (349.2 and 134.8 mosquitoes per trap-night, respectively; incidence rate ratio IRR = 1.89, 95% confidence interval CI = [1.23-2.92], P = 0.004).

The range of mosquito numbers per trap-night was 0 (in 6 and 2 trap-nights, on granitic and coralline islands, respectively) to 8,750 (in one trap with a UV light tube at Cinq Cases, Aldabra-Grande Terre, during the night of 15 Dec 2008). The number of collected adult mosquitoes per species was extremely variable.

As previously mentioned, 18 species were collected during our 2008 survey. One species was represented by a single specimen (*Cx. sunyaniensis*); two species were collected on coralline islands by the thousands (*Ae.* (*Adm.*) sp. A and *Ae. fryeri*); and the remaining 15 species (listed in Figure 2) at low or medium numbers ranging between five and a few hundred individuals. Based on a subjective impression of the authors who took part in the field survey, mosquito nuisance was undetected to low in the granitic islands and highly significant to intolerable on coralline islands.

Individual mosquito species were found at 1 to 13 sites (Table 3). The mosquito specific richness is similar for Mahé (9 sp.), Praslin (10 sp.), La Digue (10 sp.) and Aldabra (8 sp.) but much lower for Assomption (3 sp.) and Aride (1 sp.). The two collection sites with the highest specific richness are Vallée de Mai on Paslin and La Veuve Réserve on La Digue (8 sp. at each); both relictual forests within protected nature reserves.

## Discussion

Results from the present study, combined with information from the literature on the culicidian fauna of the Seychelles, highlight a number of points that deserve further attention.

### The difficulty in diagnosis of species

As usual in entomological field surveys, providing final species names for certain mosquitoes is a challenge, particularly for females with cuticular scales and setae abraded in traps. Morphology provides an invaluable contribution (especially with microscopic examinations of larva setae and/or male genitalia), although the use of gene sequencing brings considerable additional information. Combining classical morphology and molecular genetics provides a powerful set of tools to resolve alpha-taxonomic problems. In our study, these complementary analyses have demonstrated the homogeneity of gene sequences confirming that *Ae. seychellensis* is a junior synonym of *Ae. albocephalus* and resolved this long standing question. Further, we were able to solve differences in the female and larval stages of *Ae. (Adm.*) sp. A and *Ae. fryeri*.

### Specific richness and sampling effort

In examining previous entomological reports on the mosquitoes of the Seychelles and cumulative species accumulation counts, different collectors generally add no more than two species or subspecies to the Seychelles’ list. The results from our 2008 field study are in accordance with this generalization, and we added two additional species to the list of known taxa from this island group, *Cx. antennatus* and *Cx. sunyaniensis*. We also succeeded in collecting specimens of 18 resident species of Seychellois mosquitoes amongst the 21 recorded (*i.e.* the 22 species listed in Table 3 minus the non resident *An. gambiae s.l.*), a notable figure when compared to the 7 to 14 species collected during previous field studies (Table 1).

### Endemicity, vicariance- vs. dispersal-mediated divergences

**Figure 3:**
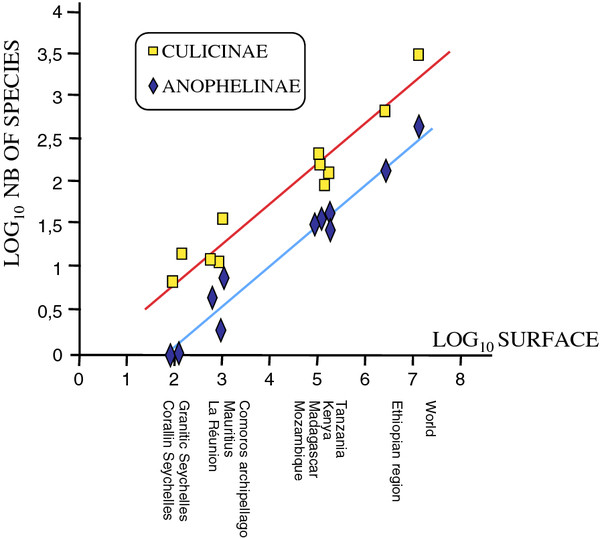
** Relationship between the mosquito specific richness and the land surface.** The number of mosquito species plotted against the land surface per country (data sources are in Additional file 2). The granitic and coralline Seychelles are considered separately. Correlation coefficient is 0.981 and 0.965 for Anophelinae and Culicinae, respectively (p < 10^-4^ in both cases).

Five mosquito species and one subspecies are thought to be endemic to the Seychelles (Table 3, see also [58]). Of these, five species or subspecies are restricted to the granitic islands and the one other species is restricted to the coralline islands. This level of endemism is accordant to the geological history of these two island types, with the granitic islands being notably older than the coralline islands. Amongst the 21 resident mosquito species, this figure of 29% endemicity (6/21) for relatively remote archipelagos and islands may seem low and might support the widely held view that mosquitoes are a group especially prone to human introduction [25,42,59]. There are currently four species suspected to have been introduced to the Seychelles (*Ae. albopictus*, *Cx. fuscocephala*, *Cx. quinquefasciatus*, *Cx. tritaeniorhynchus*) [15,34] but some of the 11 other remaining non-endemic species may also be non-native to the Seychelles. This needs to be put in perspective with regards to endemicity rates of mosquitoes within the Indian Ocean and East Africa regions. The regression plot of species richness against the log surface area of each geographic zone results in the same slopes for Anophelinae and Culicinae (Figure 3 and Additional file 2). It is noteworthy that such correlations are in agreement with the theory of island biogeography [60,61]. 

### The competition between *Ae. albopictus* and *Ae. aegypti*

*Aedes albopictus* has traditionally been considered as a non-invasive species in the Seychelles based on different measures of relative abundance since, at least, the beginning of the 20^th^ century. However, the introduction by marine vessels of this species in the Seychelles has long been suspected. Accordingly, based on the fieldwork of Harper [24] and Brown [25], the continued decrease in the relative density of *Ae. aegypti* on the granitic islands during the second half of the 20^th^ century reinforces the hypothesis that a new population of *Ae. albopictus* with invasive behaviour was introduced in the 1950s. *Aedes albopictus* is a voracious biter. It now constitutes the main diurnal arthropod nuisance on the granitic islands, biting at all times of day in the deep shade of forest and plantation, as well as in urban areas. The current abundance of *Ae. aegypti* on the granitic islands is quite anecdotal but this situation has developed only recently. During the mid 20^th^ century, *Ae. albopictus* was considered as a rural mosquito and *Ae. aegypti* being more closely dependant of anthropogenic habitat. Lambrecht [15] hypothesized the following, “As long as the Seychelles environment remains essentially rural, *Ae. albopictus* will probably remain the predominant species, giving way to *Ae. aegypti* in areas of high human densities.” Perhaps due to changes in the genetic background of *Ae. albopictus* in the Seychelles during the second half of the 20^th^ century, this prediction, although put forward by an eminent entomologist, did not materialize.

Subsequent field studies underlined the limited distribution of *Ae. aegypti* on Mahé [6], including only three *breeding-sites* in a large survey amongst the residential areas of Mahé (among which two co-breeding with *Ae. albopictus*) [21]. Accordingly, our study did not observed any *Ae. aegypti* on the granitic islands. The situation on these islands is now clear — *Ae. albopictus* has won the interspecific competition with *Ae. aegypti*. More exhaustive surveys are needed to monitor if this competitive exclusion is absolute or partial, with a small population of *Ae. aegypti* succeeding to survive, as is presently the case on La Réunion [44]. This same situation, with *Ae. albopictus* replacing *Ae. aegypti*, is well documented elsewhere in the world [48-50,62,63].

By contrast, *Ae. albopictus* seems absent from the coralline islands in the Aldabra group, where *Ae. aegypti* is abundant. This observation would support the hypothesis of competitive exclusion of the latter by the former. These remote islands, with no true harbour and almost completely uninhabited, may be at relatively low risk of introduction of *Ae. albopictus*. Most probably, this species has been introduced in these islands on occasion through human intervention, but without succeeding to establish permanent functional populations. While this is pure speculation, the notable plasticity of *Ae. aegypti* in terms of hosts it feeds upon [64], including birds and reptiles (including Aldabra tortoises), may provide a major adaptive advantage on these coralline islands where the only non-introduced mammals are bats [1].

### Most notable mosquito aggressiveness on coralline islands

On the coralline islands, during the rainy season, the nuisance was considerable to unbearable without protective measures (repellents, long clothes, net, etc.). Mosquito aggressivity towards humans was almost equivalent to those observed during the rainy season on Europa Island [65] and during summer in boreal countries such as northern Canada and Scandinavia (Robert, pers. obs.). In the case of the Seychelles coralline islands where native mammals are almost absent, this observation is puzzling. In fact, native bats and few introduced mammals (rats, cats, goats) exist on coralline islands, although it is very likely that these mammals do not constitute a significant source for blood feeding. We suspect that most blood meals are probably taken from birds, which are abundant, and reptiles, especially Aldabra tortoises (their population is estimated at c. 100,000 on Aldabra). It was common to observe mosquitoes biting the legs and necks of these tortoises and the relative thickness of their tegument is not an obstacle for mosquitoes. When possible, tortoises were observed to immerse themselves in water pools during the night. The massive number of mosquitoes searching for blood meals may drive this behaviour. No haemoparasites have been observed in the blood of 78 Aldabra tortoises [66] and in 143 feral Aldabra tortoises living on Curieuse [67], although some haemoproteids have been found in South African terrestrial tortoises [68]. Because mosquitoes equally bite the marine turtles when they come ashore to lay, it is possible that mosquitoes transmit infectious agents between marine turtles and land tortoises.

### Introduction followed or not by subsequent colonization

The different mosquito populations that succeed in reaching the Seychelles have clearly met different fates. Some, such as those belonging to the *An. gambiae* complex arrived to the Aldabra group, with a mention of a outbreak of human malaria in 1930, followed by an apparent total extinction during the first subsequent dry season [1,17]. On the other hand, *Cx. fuscocephala*, which was observed for the first time in 1995 [21], and probably of eastern Indian Ocean origin, is now firmly established on the granitic islands, even if the population size seems low.

## Conclusion

The mosquito fauna of the Seychelles currently comprises 22 species, among which six taxa (five species and one subspecies) are endemic to this country. However, if we consider only species that have established reproductive populations, the resident culicidian fauna is composed of 21 species, all belonging to Culicinae subfamily. The absence of any resident anopheline mosquito, the vector of malaria, is unique in this part of the world; consequently the Seychelles is malaria free.

Amongst these 21 species, our survey in December 2008 found 18 species, including two species new for the entomological fauna of the Seychelles, namely *Cx.* (*Cux.*) *antennatus* and *Cx.* (*Eum.*) *sunyaniensis*.

*Aedes seychellensis* is placed as a junior synonym of *Ae. (Adm.) albocephalus.*

The mosquito species of these islands provide numerous examples of events such as introduction, invasion, colonization and extinction, giving superb illustrations for theoretical and applied island biogeography.

Additional studies on the biogeography and ecology of Seychelles mosquitoes are highly desirable, in particular on the relationships between historical and environmental factors, species richness and abundance of Culicidae on the different islands. Overall impacts of mosquito vectors on public health and the country’s economy, but also on wildlife conservation, deserve further investigation; this includes how to minimize the negative impacts of the species currently present and the risks of new introductions of alien invasive mosquitoes.

## Competing interests

The authors declare that they have no competing interests.

## Authors’ contributions

GR and VR motivated and designed the study, and acquired funding for the field survey. SJ, GR and VR performed the fieldwork. GLG, PB and VR performed the bibliographic study and the identification of mosquito species. CB performed the molecular biology work and gene sequencing. GLG and PB made the keys. PB and NR drew the plates for the keys. VR wrote the first draft version. All authors worked on successive drafts and approved the final version of the manuscript.

## Supplementary Material

Additional file 1 ** Morphological key to adult females of the 22 mosquito species found at least once in the Seychelles.** - Morphological key to larval stage IV of the 21 mosquito species found at least once in the Seychelles. - Plate captions- Plates 1 and 2. Adult female mosquitoes of the Seychelles. - Plates 3 and 4. Larval mosquitoes of the Seychelles. Click here for file

Additional file 2 The species richness of resident mosquitoes in portions of the south-west Indian Ocean. Click here for file
