# Supplementary material for: The mosquitoes (Diptera: Culidae) of Seychelles: taxonomy, ecology, vectorial importance, and identification keys
Source: Parasit Vectors. 2012 Sep 21;5:207. doi: 10.1186/1756-3305-5-207 (PMC3484020; doi:10.1186/1756-3305-5-207)
Supplement: Additional file 1 — Morphological key to adult females of the 22 mosquito species found at least once in the Seychelles. - Morphological key to larval stage IV of the 21 mosquito species found at least once in the Seychelles. - Plate captions- Plates 1 and 2. Adult female mosquitoes of the Seychelles. - Plates 3 and 4. Larval mosquitoes of the Seychelles. [file 1756-3305-5-207-S1.pdf]

Le Goff G, Boussès P, Julienne S, Brengues C, Rahola N, Rocamora G & Robert V - The mosquitoes (Diptera: Culidae) of Seychelles: taxonomy, ecology, vectorial importance, and identification keys. *Parasites & Vectors* 2012. Additional file 1. (10 printed pages, including 4 plates)

Morphological key to adult females of the 22 mosquito species found at least once in the Seychelles

Morphological terms in this key are after Harbach and Knight, 1980 [67].

1.	- Maxillary palpus as long as the proboscis [Plate 1 a.1], wing usually spotted<br>- Other cases [Plate 1 a.2]	*Anopheles gambiae s.l.*<br>2
2.	- Wing with anal vein (1A) ending near the termination of the fork between cubitus anterior vein (CuA) and mediocubital crossvein (mcu) [Plate 1 b.]; mosquito of small size (2.5-4 mm)<br>- Wing with anal vein (1A) ending beyond the termination of the fork between cubitus anterior vein (CuA) and mediocubital crossvein (mcu) [Plate 1 c.]; mosquito of medium size (4.5-7 mm)	Genus *Uranotaenia* 3<br>5
3.	- Terga fully dark, without basal bands<br>- Terga with basal bands, especially including terga IV to VI	*Ur. browni*<br>4
4.	- Postnotum of varying color, fully brown or with a median yellowich band; terga I, II and III unbanded; terga IV-VI more or less distinctly banded<br>- Postnotum fully light and yellowich; distinct yellowish basal bands on terga III-VI (not on terga I and II)	*Ur. pandani*<br>*Ur. nepenthes*
5.	- Presence of postspiracular setae [Plate 1 d.]<br>- Postspiracular setae absent	6<br>Genus *Culex* 12
6.	- Veins of the wing only covered with a mixture of large scale black and white [Plate 1 e.]; abdomen with obtuse apex; reduced 7th abdominal segment; legs with simple claws [Plate 2 b.1]<br>- Wing not so; abdomen with pointed apex; 7th abdominal segment of normal size; legs with dentate claws [Plate 2 b.2]	*Mansonia uniformis*<br>Genus *Aedes* 7
7.	- Hindtarsus [Plate 2 a.] with ≥ 2 tarsomeres with rings of light scales<br>- Hindtarsus fully dark, or with a single small ring or spot of light scales on hindtarsomere I	8<br>11
8.	- Proboscis fully dark [Plate 1 a.2]<br>- Proboscis partly light [Plate 2 c.]	*Aedes* subgenus *Stegomyia* 9<br>10
9.	- Scutum with lyre-shaped white marking; clypeus with white scale-patches<br>- Scutum without lyre-shaped white marking; clypeus without white scale-patches	*Ae. aegypti*<br>*Ae. albopictus*
10.	- Terga VI and VII mainly dark in the middle segments (antero-lateral white spots present) [Plate 2 d.1]<br>- Terga VI and VII with white scales delineating a median, large and continuous band (in addition of the 2 lateral white spots) [Plate 2 d.2]<br>- Terga VI and VII with white scales delineating a median spot on the basal part only band (in addition of the 2 lateral white spots) [Plate 2 d.3]	*Ae.* (*Adm.*) sp A<br>*Ae. fryeri*<br>*Ae. vigilax vansomerenae*

11.	- Hindtibia with white apical spot; lower mesepimeral seta absent<br>- Hindtibia without white apical spot; lower mesepimeral seta present (only in females)	*Ae. albocephalus*<br>*Ae. lambrechti*
12.	- Abdominal terga with basal transverse pale bands [Plate 2 e.1]<br>- Abdominal terga without basal transverse pale bands [Plate 2 e.2 and e.3]	13<br>17
13.	- Presence of 1 or 2 (exceptionally 3) lower mesepimeral setae; proboscis without distinct ring of light scales (proboscis dark scaled on dorsal surface and sometimes paler on lateral and ventral surfaces); tarsomeres fully dark, without any basal and apical light bands<br>- Lower mesepimeral setae absent; proboscis with a complete median ring of light scales; tarsi with light bands on basal and/or apical parts	14<br>16
14.	- Presence of postspiracular and prealar scales [Plate 2 f.]<br>- Postspiracular and prealar scales absent	*Cx. simpsoni*<br>15
15.	- Sternum all pale, without band; ventral proboscis light<br>- Sternum banded, with dark apical bands; proboscis fully dark	*Cx. quinquefasciatus*<br>*Cx. scottii*
16.	- Anterior surface of fore and midfemora with speckling of several pale scales at least on apical dorsal surface; scales on all wing veins entirely dark; wing with a short R2 cell, the fork of the veins R2-R3 (cell R2) being closer to the wing apex than the fork of the veins M1-M2 (cell M1)<br>- Anterior surface of fore and midfemora entirely dark; costa with a small spot of pale scales at base; wing with a R2 cell of large, size cell R2 4, 5 times as long as R2+3, the fork of the veins R2-R3 (cell R2) being not closer to the wing apex than the fork of the veins M1-M2 (cell M1)	*Cx. sitiens*<br>*Cx. tritaenyorhynchus*
17.	- With broad lateral stripes of cream-coloured scales on abdominal terga VI and VII [Plate 2 e.2]<br>- Without such lateral stripes on terga VI and VII [Plate 2 e.3]	*Cx. antennatus*<br>18
18	- Presence of lower mesepimeral setae<br>- No lower mesepimeral setae	20<br>*Cx. stellatus*
20.	- Pleuron with striking pattern of dark and pale stripes, pleural scale patches present, usually 2 on mesokatepisternum (Mks) and 1 on mesepimeron (Mam)<br>- Pleuron without striking pattern of dark and pale stripes. No patch of white scales on mesokatepisternum	*Cx. fuscocephala*<br>21
21	- Vertex with a large spot of white erect scales [Plate 2 c.]<br>- Vertex with a small number of white erect scales	*Cx. wigglesworthi*<br>*Cx. sunyaniensis*

Morphological key to larval stage IV of the 21 mosquito species found at least once in the Seychelles

The larva of *Cx. scottii* is unknown, but Lambrecht assumed that "atypical *Cx. quinquefasciatus*" larva might belong in fact to *Cx. scottii* [13]. The larva of *Ae. (Sku.) lambrechti* was not collected during our field survey, the types of this species were lost in transit to the British Museum in 1975, and the paratypes do not include larvae (Miss Theresa Howard, pers. com.); therefore the key below uses the original text description [33]. The larva of *Ae. vigilax vansomerene* was not collected during our field survey, and cuplet 11 (distinction between *Ae. vigilax vansomerenae* and *Ae. fryeri*) must be considered with some caution, keeping in mind that the former had only been found on the granitic islands and the latter on the coralline islands.
Morphological terms in this key are after Harbach and Knight, 1980 [67].

1.	- Absence of siphon [Plate 3 a.1]	*Anopheles gambiae s.l.*
	- Siphon attenuated, sclerotinized saw-toothed projection at tip, modified for piercing plant tissues [Plate 3 a.2]	*Mansonia uniformis*
	- Siphon cylindrical or spindle shaped, not attenuated [Plate 3 a.3]	2
2.	- Siphon with single pair of sub-ventral seta 1-S [Plate 3 b.1]	3
	- Siphon with sub-ventral seta 1-S more numerous [Plate 3 b.2]	Genus *Culex* 12
3.	- Hypostomal suture absent or incomplete; head longer than broad [Plate 3 c.1]; usually, a large lateral chitinous plate present on abdominal segment 8	Genus *Uranotaenia* 4
	- Hypostomal suture reaching posterior tentorial pit; head broader than long [Plate 3 c.2]; abdominal segment 8 without large lateral chitinous plate	Genus *Aedes* 6
4.	- Thorax and abdomen with many stellate setae; siphon with many long spines [Plate 3 d.]	*Ur. browni*
	- Thorax and abdomen with stellate setae; siphon with no or few long spines	5
5.	- Abdomen with lateral setae of first six segments (6-I-VI) fine and pale; comb scales denticulate [Plate 3 e.1]	*Ur. pandani*
	- Abdomen with lateral setae of first six segments (6-I-VI) long, stout and dark; comb scales fringed [Plate 3 e.2]	*Ur. nepenthes*
6.	- Antenna smooth or very thinly spiculate [Plate 3 f.1]; seta 4-X inserted only on the barred area [Plate 3 g.1]	7
	- Antenna spiculate, sometimes lightly spiculate [Plate 3 f.2]; ventral tufts of which 1-4 arise outside the barred area [Plate 3 g.2]	9
7.	- Comb with a patch of more than 50 scales; siphon cylindrical and pale, about 3.5 times as long as broad; [Plate 3 g.1]	*Ae. lambrechti*
	- Comb with a single curved row of less than 20 scales, siphon not cylindrical; [Plate 3 g.2]	8
8.	- A strong curved and sharply-pointed spines at base of setae 9-12 of meso and metathorax; comb scales with strong and prominent basal denticules [Plate 3 h.1]; ventral brush 4-X is 2 branched setae [Plate 3 h.2]	*Ae. aegypti*
	- Small spines at base of setae 9-12 of meso and metathorax; comb scales without strong basal denticules [Plate 3 h.3]; Ventral brush 4-X of single setae [Plate 3 h.4]	*Ae. albopictus*

9.	- Head setae 5-C and 6-C are 5-6 branched [Plate 3 i.1]	*Ae. albocephalus*
	- Head setae 5-C and 6-C are 1-2 branched [Plate 3 i.2]	10
10.	- Comb with < 15 scales; anal papilla spindled as long as 8/10 of the saddle [Plate 3 g.2]	*Ae. (Adm.)* sp A
	- Comb with ≥ 15 scales; anal papilla near spherical, much shorter than the saddle [Plate 4 a.1 and a.2]	11
11.	- The body of the comb scale with a median denticule stouter than the remainder [Plate 4 a.1]	*Ae. fryeri*
	- The body of the comb scale frequently drawn into a point apically but the median denticule not noticeably stouter than the remainder [Plate 4 a.2]	*Ae. vigilax vansomerenae*
12.	- Thorax and abdomen with stellate setae [Plate 4 b.]	*Cx. stellatus*
	- Setae single, branched, not stellate	13
13.	- Comb mostly composed of spine-like scales [Plate 4 c.1]	*Cx. simpsoni*
	- Comb only composed of fringed scales [Plate 4 c.2]	14
14.	- Head setae 5-C and 6-C branched, usually 5-6 branched (5-9) [Plate 4 d.1]	15
	- Head setae 5-C and 6-C usually double or triple (1- 4 branches) [Plate 4 d.2]	16
15.	- Seta 1-C long and slender [Plate 4 e.3]; pecten teeth with 2- to 4-denticules [Plate 4 e.2]; anal gills as long as the saddle of the anal segment or longer [Plate 4 e.1]	*Cx. quinquefasciatus*
	- Seta1-C thick, stout and sometimes spiculate at side [Plate 4 e.6]; pecten teeth with many denticules [Plate 4 e.5]; anal gills very short [Plate 4 e.4]	*Cx. sitiens*
16.	- Siphon index ≤ 6	17
	- Siphon index ≥ 8	18
17.	- Setae l-IV and 1-V of abdomen are single and as longer than length of 2 abdominal segments [Plate 4 f.1]	*Cx. fuscocephala*
	Setae l-IV and 1-V are shorter than length of 2 abdominal segments, with 2-3 or more branches [Plate 4 f.2]	*Cx. antennatus*
18.	- Ventral brush (seta 4-X0) without precratal setae outside the barred area [Plate 3 a.3]	*Cx. tritaeniorhynchus*
	- Ventral brush with precratal setae outside the barred area [Plate 4 g.1 and g.2]	19
19.	- Siphon and saddle dark; seta 1-S lower than the diameter of the siphon; segment X without spiculate apical margin [Plate 4 g.1]	*Cx. sunyaniensis*
	- Siphon and saddle pale; seta 1-S slightly longer than the diameter of the siphon; segment X with spiculate apical margin [Plate 4 g.2]	*Cx. wigglesworthi*

Plate captions

PLATE 1: Adult female mosquitoes of the Seychelles

a. Head of Culicinae spp. (figure on the left) and head of Anophelinae spp. (right) showing important structures: Ant, antenna; CE, compound eye; Clp, clypeus; MPlp, maxillary palpus; Occ, occiput; P, proboscis; Pe, pedicel; V, vertex

b. Wing of *Uranotaenia* spp. showing venation and cells (cells are underlined): Al, alula; C, costa; CuA, cubitus anterior; h, humeral crossvein; LC, lower calypter; M1, media one cell; M1, media one; M2, media two; M3+4, media three plus four; m-cu, mediocubital crossvein; r-m, radiomedial crossvein; R1, radius one; R2, radius two cell; R2, radius two; R2+3, radius two plus three; R3, radius three; R4+5, radius four plus five; Sc, subcosta; UC, upper calypter; WF, wing fringe; 1A, anal cell; 1A, anal vein.

c. Wing of Culicinae spp. other than *Uranotaenia* spp. Abbreviations idem Plate I b.

d. Thorax of an *Aedes* spp. male (left lateral view): A, abdomen; Ap, antepronotum; C-I, forecoxa; C-II, midcoxa; C-III, hindcoxa; H, head; Hl, halter; Mam, mesanepimeron; MeSL, lower mesepimeral seta; MeSU, upper mesepimeral seta; Mks, meskatepisternum; Mpn, mesopostnotum; MS, mesothoracic spiracle; PA, postspiracular area; Pa, paratergite; PaS, prealar seta; Pn, postnotum; Ppn, postpronotum; PS, postspiracular setae; Scu, scutum; Stm, scutellum; W, wing.

e. Detail of a *Mansonia* spp. wing showing the mixture of large black and white scales on the radius vein 3.

PLATE 2: Adult female mosquitoes of the Seychelles (continuated)

a. Fore leg of Culicinae spp.

b. Hind tarsal claw dentate (figure on the left) and simple (right).

c. Head of *Culex* spp. showing the proboscis partly light and the erect forked scales of the vertex (V).

d. Abdomens of *Aedes* spp. (dorsal view): *Aedes* (*Aedimorphus*) sp. A (1); *Aedes fryeri* (2); *Aedes vigilax vansomerenae* (3); *Aedes fowleri* (4). *Aedes fowleri* although not recorded in the Seychelles is presented given its broad distribution in the Afrotropical region, including islands of the south-west Indian Ocean.

e. Abdomens of *Culex* spp. (dorsal view) showing basal transverse pale bands (1) and other ornamentations (2 and 3)

f. Thorax of *Culex* spp. (left lateral view) showing some scales locations: A, abdomen; H, head; Hl, halter; Mam, mesanepimeron; Mks, meskatepisternum; MS, mesothoracic spiracle; PoSc, postspiracular scale; PrSc, upper and lower prealar scale; W, wing.

PLATE 3: Larval mosquitoes of the Seychelles

a. Lateral schematic view of segment VIII of Anophelinae spp. (1), *Mansonia* spp. (2) and Culicinae spp. (3) (here *Culex simpsoni*).

b. Lateral view of siphon of *Aedes* spp. (1) and *Culex quinquefasciatus* (2) with indication of the seta 1-S.

c. Ventral view of the head of *Aedes* spp. (1) and *Uranotaenia pandani* (2).

d. Schematic dorsal view of the thorax of *Uranotaenia browni*.

e. Comb scales of *Uranotaenia pandani* (1) and *Uranotaenia nepenthes*.

f. Antenna not spiculated of *Aedes* sp. (1) and spiculated of *Aedes* sp. A (2).

g. Lateral view of posterior abdomen of *Aedes lambrechti* (1) and *Aedes* sp. A.

h. Lateral view of abdominal segment X and comb scales of *Aedes aegypti* (1 and 2) and *Aedes albopictus* (3 and 4).

i. Dorsal view of the head of *Aedes albocephalus* (1) and *Aedes* sp. A (2).

PLATE 4 : Larval mosquitoes of the Seychelles (continued)

a. Lateral schematic view of posterior abdomen of *Aedes fryeri* (1) and *Aedes vigilax vansomerenae* (2).

b. Dorsal view of head and thorax of *Culex stellatus*.

c. Comb scales of *Culex simpsoni* (1) and *Culex quinquefasciatus* (2).

d. Dorsal view of head of *Culex* spp.

e. Lateral view of abdominal segment X of *Culex quinquefasciatus* (1) with comb scales (2) and dorsal view of the left-anterior quarter of the head (3); and *Culex sitiens* (4, 5 and 6).

f. Dorsal view of the abdominal segments IV to VI of *Culex* spp with setae 1 long and simple (1) and short and multiple (2).

g. Lateral schematic view of posterior abdomen of *Culex sunyaniensis* (1) and *Culex wigglesworthi* (2).

a.

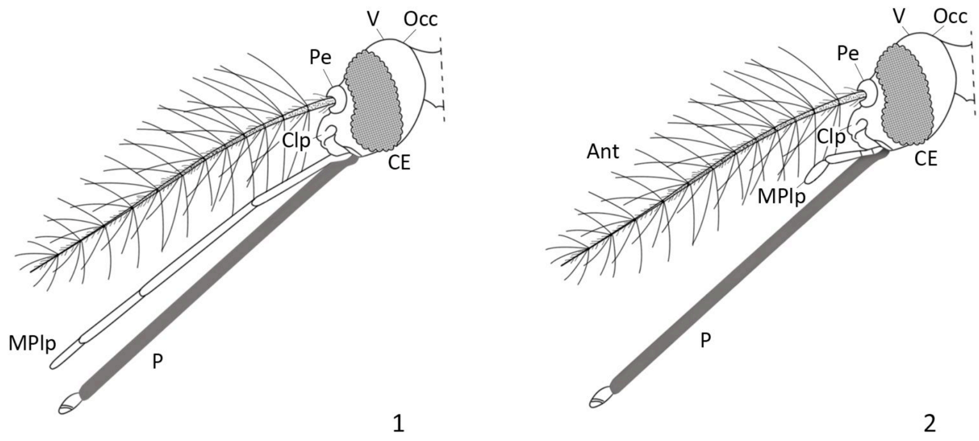

b.

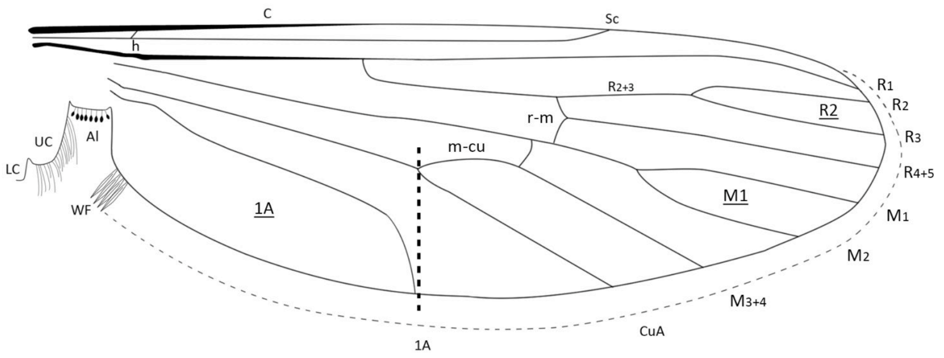

c.

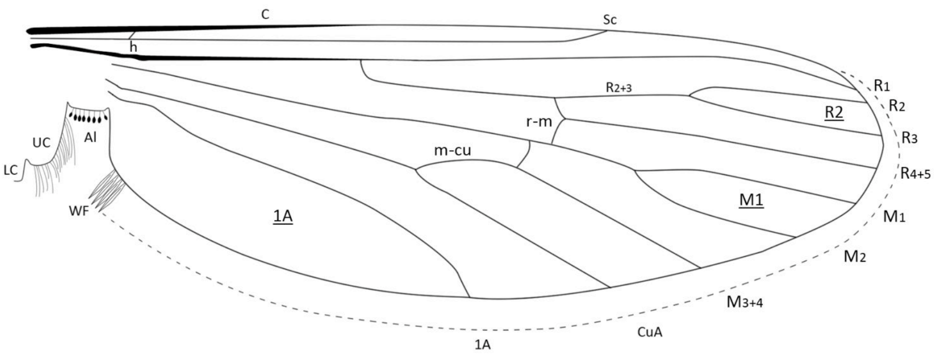

d.

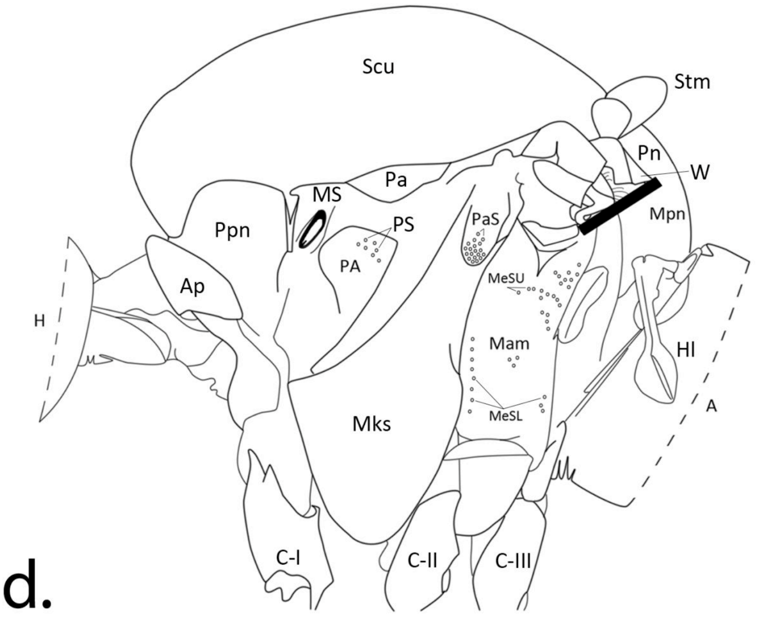

e.

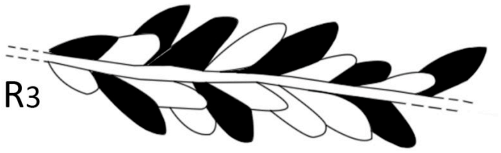

PLATE 1

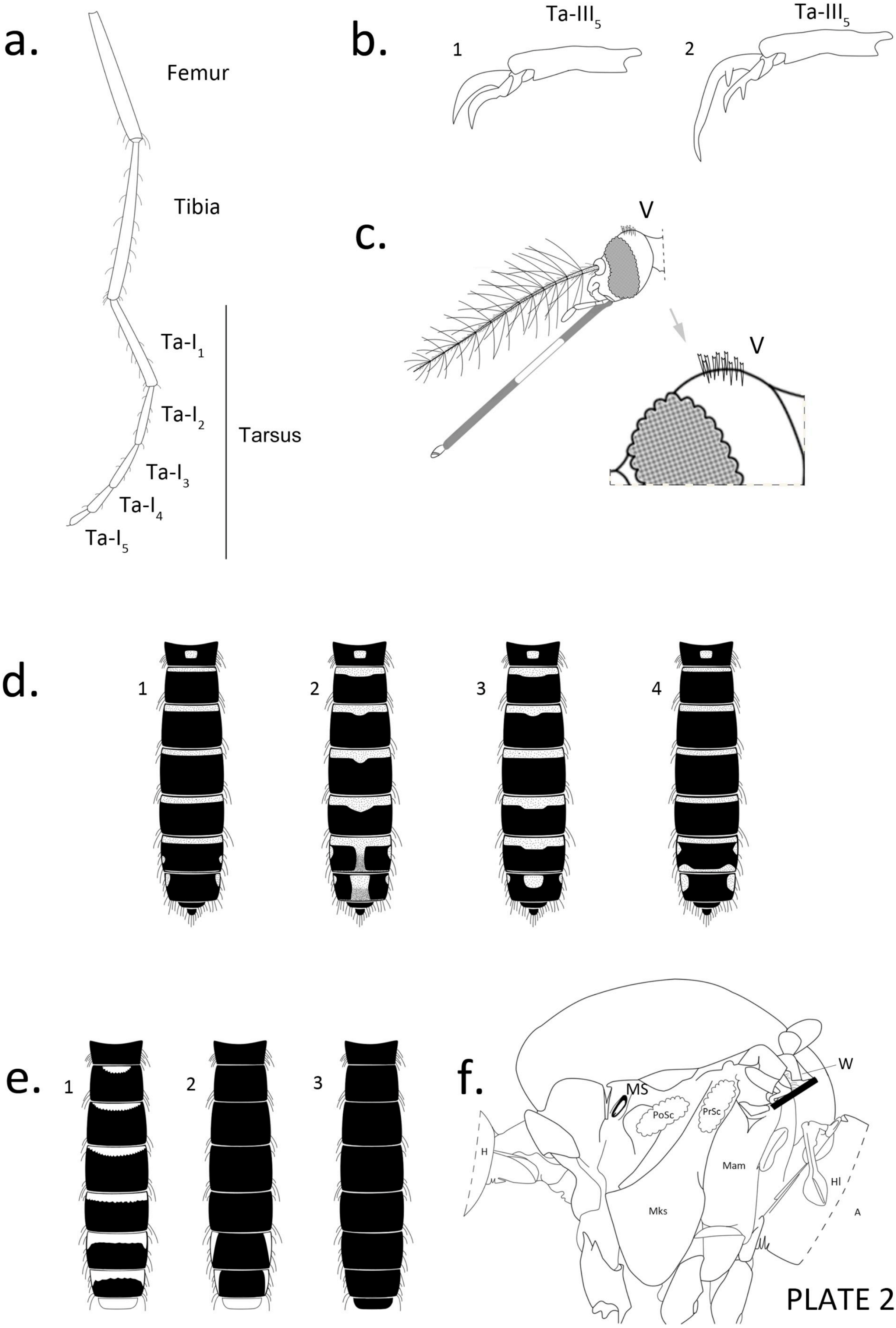
a.
Femur
Tibia
Ta-I$_1$
Ta-I$_2$
Ta-I$_3$
Ta-I$_4$
Ta-I$_5$
Tarsus
b.
1
Ta-III$_5$
2
Ta-III$_5$
c.
V
V
d.
1
2
3
4
e.
1
2
3
f.
W
MS
PoSc
PrSc
H
Mam
Mks
Hl
A

PLATE 2

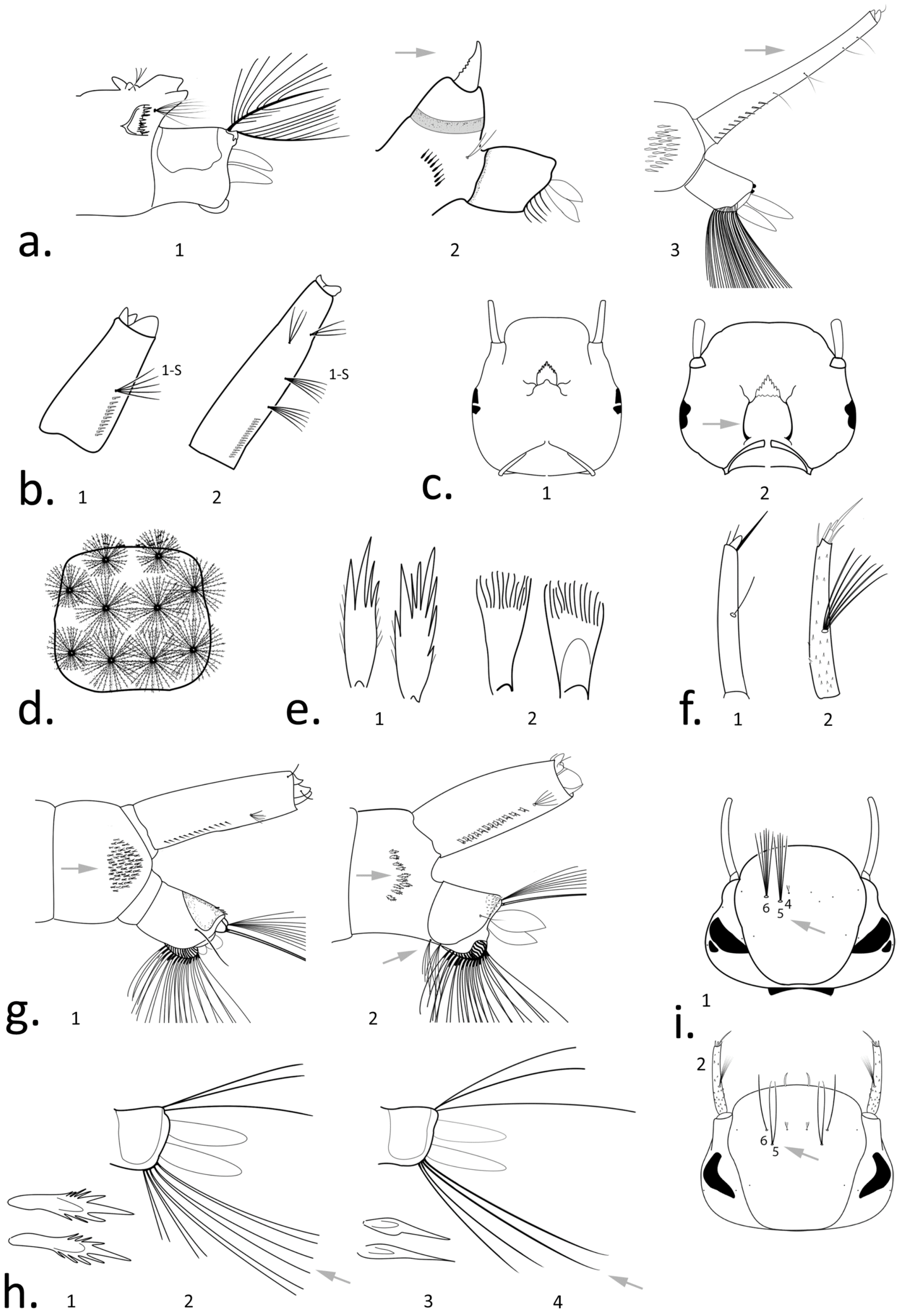

PLATE 3

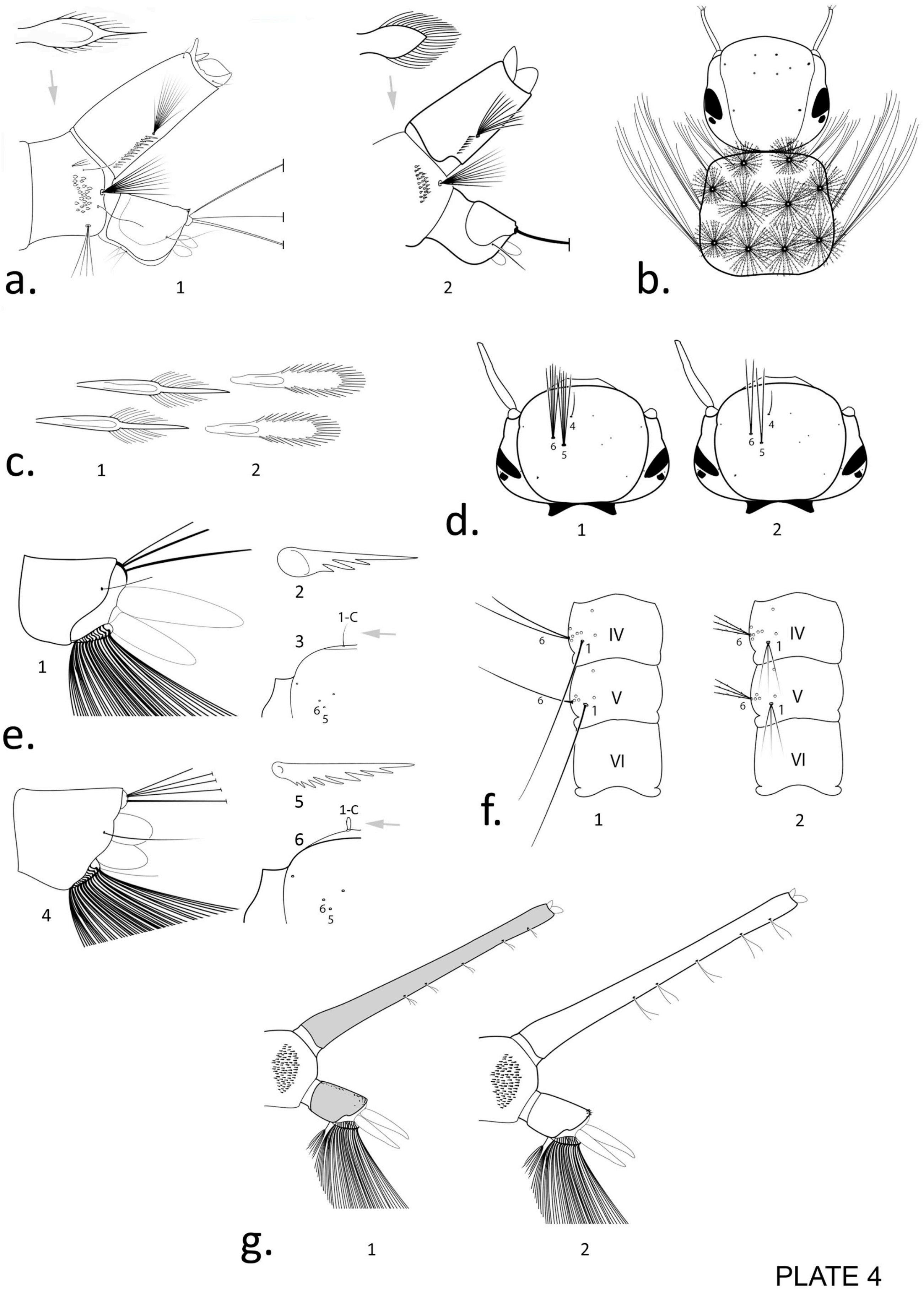

PLATE 4
