# Supplementary material for: The mosquitoes (Diptera: Culidae) of Seychelles: taxonomy, ecology, vectorial importance, and identification keys
Source: Parasit Vectors. 2012 Sep 21;5:207. doi: 10.1186/1756-3305-5-207 (PMC3484020; doi:10.1186/1756-3305-5-207)
Supplement: Additional file 2 — The species richness of resident mosquitoes in portions of the south-west Indian Ocean. [file 1756-3305-5-207-S2.pdf]

The species richness of resident mosquitoes in portions of the south-west Indian Ocean

This additional file presents the data for drawing the Figure 3.

The data sources for species numbers is derived from WRBU (www.mosquitocatalog.org/) that were updated according to published literature. Introduced species that did not succeed to establish permanent functional population(s) (*i.e.* failed early in the colonization process) were not considered. Subspecies were also not considered.

	Anophelinae		Culicinae	
	Nb of species	Nb of restricted range species	Nb of species	Nb of restricted range species
World	478	178 (36%)	3043	1466 (48%)
Ethiopian region [a]	142	53 (37%)	662	292 (44%)
Kenya	41	7 (10%)	173	27 (11%)
Tanzania	48		129	
Mozambique	29		112	
Madagascar	26	11 (42%)	212	123 (58%)
Comoros archipelago	8	1 (12%)	34	6 (18%)
La Réunion	2	0	10	1 (10%)
Mauritius	4	0	13	4 (31%)
Granitic Seychelles	0	0	16	5 (31%)
Coralline Seychelles	0 [b]	0	8	1 (13%)

[a] Only for species rank; Yemen and Oman excluded
[b] *An. gambiae* excluded as non-resident mosquito
